# Establishing key components of yoga interventions for musculoskeletal conditions: a Delphi survey

**DOI:** 10.1186/1472-6882-14-196

**Published:** 2014-06-18

**Authors:** Lesley Ward, Simon Stebbings, Karen J Sherman, Daniel Cherkin, G David Baxter

**Affiliations:** 1 Centre for Health, Activity and Rehabilitation Research, School of Physiotherapy, University of Otago, Dunedin, New Zealand; 2 Department of Medicine, Dunedin School of Medicine, University of Otago, Dunedin, New Zealand; 3 Group Health Research Institute, Seattle, Washington, USA

**Keywords:** Yoga, Musculoskeletal conditions, Clinical trials, Guidelines, Delphi

## Abstract

**Background:**

Evidence suggests yoga is a safe and effective intervention for the management of physical and psychosocial symptoms associated with musculoskeletal conditions. However, heterogeneity in the components and reporting of clinical yoga trials impedes both the generalization of study results and the replication of study protocols. The aim of this Delphi survey was to address these issues of heterogeneity, by developing a list of recommendations of key components for the design and reporting of yoga interventions for musculoskeletal conditions.

**Methods:**

Recognised experts involved in the design, conduct, and teaching of yoga for musculoskeletal conditions were identified from a systematic review, and invited to contribute to the Delphi survey. Forty-one of the 58 experts contacted, representing six countries, agreed to participate. A three-round Delphi was conducted via electronic surveys. Round 1 presented an open-ended question, allowing panellists to individually identify components they considered key to the design and reporting of yoga interventions for musculoskeletal conditions. Thematic analysis of Round 1 identified items for quantitative rating in Round 2; items not reaching consensus were forwarded to Round 3 for re-rating.

**Results:**

Thirty-six panellists (36/41; 88%) completed the three rounds of the Delphi survey. Panellists provided 348 comments to the Round 1 question. These comments were reduced to 49 items, grouped under five themes, for rating in subsequent rounds. *A priori* group consensus of ≥80% was reached on 28 items related to five themes concerning defining the yoga intervention, types of yoga practices to include in an intervention, delivery of the yoga protocol, domains of outcome measures, and reporting of yoga interventions for musculoskeletal conditions. Additionally, *a priori* consensus of ≥50% was reached on five items relating to minimum values for intervention parameters.

**Conclusions:**

Expert consensus has provided a non-prescriptive reference list for the design and reporting of yoga interventions for musculoskeletal conditions. It is anticipated future research incorporating the Delphi guidelines will facilitate high quality international research in this field, increase homogeneity of intervention components and parameters, and enhance the comparison and reproducibility of research into the use of yoga for the management of musculoskeletal conditions.

## Background

Musculoskeletal conditions comprise a diverse group of over 200 disorders affecting muscle, bone, and cartilage [1-3]. While a heterogeneous group in terms of their classification, pathophysiology, and clinical presentation, these conditions share important features including pain, functional disability, and decreased quality of life [2,4-6]. Collectively, musculoskeletal conditions are a major health and economic burden, both in developed and developing countries [3,7-10].

The current consensus for best practice in the management of musculoskeletal conditions is to modify symptoms, limit disease progression and functional limitation, and improve health-related quality of life [3,11,12]. Current clinical guidelines recommend a combination of pharmacological and non-pharmacological therapies [11-15]. An increasingly popular form of non-pharmacological therapy for the management of musculoskeletal conditions is the mind-body therapy of yoga [16,17].

The practice of yoga was first recorded in India over 4000 years ago, as a spiritual practice for uniting the body, mind, and emotions [18-21]. As knowledge of yoga has disseminated from India into different countries, the practice has moved away from this purely spiritual focus, and in the West yoga is increasingly practised as a form of exercise [22], and as a health therapy [16,19]. The most common form of yoga in the West is Hatha yoga, a combination of physical, breathing, and mental practices [20,23,24]. Within Hatha yoga, there are a variety of styles, differing in the emphasis they place on these different yoga practices [25,26]. These include alignment-based styles such as Iyengar yoga [20], physically vigorous styles such as Bikram yoga [27], and relaxation-based styles such as restorative yoga [28].

Evidence suggests that yoga has a moderate effect on pain and functional outcomes across a range of musculoskeletal conditions [29-31]. However, results are tempered by the substantial heterogeneity associated with these intervention studies. In the context of clinical research, mind-body therapies such as yoga may be defined as complex interventions, characterised by multiple components capable of being delivered in numerous combinations [32,33]. This multi-component, multi-interactive nature of a yoga intervention presents methodological challenges in the context of clinical research, such as defining the content and delivery of these interventions [34-36].

Currently, no recommendations exist for the content and reporting of clinical yoga interventions for musculoskeletal conditions. For complex, multi-component therapies such as yoga, this lack of guidance has resulted in trials weakened by heterogeneity of design components and challenges in replication of study protocols [25,37]. To address these issues, standardisation of yoga interventions has been proposed [25,37-39]. The main challenges with this proposal are to determine which intervention components are amenable to standardisation [35,36]; and to balance this need for standardisation with the ability to adapt an intervention to the specific needs of the clinical population, the outcome measures being studied, and the style of yoga being delivered [40].

The aim of this study was to develop a list of Delphi recommendations of key components for the design and reporting of yoga interventions for musculoskeletal conditions. This study is reported in accordance with reporting guidelines for Delphi surveys [41,42].

## Method

As the geographical diversity of research teams involved in evaluating yoga for musculoskeletal conditions precludes face-to-face interaction, establishing international recommendations for standardisation of these interventions requires participants to interact without the necessity of a physical meeting [43]. To overcome this challenge, we used the Delphi technique, which is increasingly used in healthcare research [44-46]. A survey-based method of consensus building, the Delphi technique is based on fundamental principles of purposive sampling of experts in the field of interest, panellist anonymity, iterative questionnaire presentation, and feedback of statistical analysis [41,42,47-49].

The development process comprised a survey-based modelling process [35,36], using the Delphi technique. Identified experts in the research field completed three rounds of a Delphi survey, identifying, then rating, key intervention components. Items reaching *a priori* consensus were included in the resultant list of Delphi recommendations. The conduct of the Delphi survey was guided by previous reviews of the Delphi method [41,42,48-51]. Criteria for analysis, consensus, and termination reflect those of past Delphi surveys in the field of health research and complementary medicine (e.g. [43,45,46,52-54]). Ethical approval for the study was granted by the University of Otago Ethics Committee.

A five-member Steering Committee, chaired by LW, oversaw the conduct of the Delphi survey. Members included musculoskeletal researchers, yoga teachers, a rheumatologist, and a physiotherapist. The Steering Committee determined *a priori* criteria for item consensus and survey termination, questionnaire development, and data analysis [52,54].

### Selection and recruitment of expert panellists

A systematic review of yoga for musculoskeletal conditions was conducted [31], to identify experts in the research field [48,55]. ‘Experts’ were operationalized as individuals involved in the conception, design, conduct, teaching, or analysis of yoga interventions for musculoskeletal conditions. These broad criteria were designed to ensure a range of research stakeholders were included in the Delphi survey [36,56].

Fifty-eight experts received personalised emails inviting them to participate in the Delphi survey. Invitations included an information pack highlighting proposed dates and time commitments for the survey. Additionally, a snowball technique enabled experts to recommend others who met the inclusion criteria but may not have been identified by the systematic review. Non-responders to the invitation received a follow-up email three weeks later, reporting confirmed numbers of panellists recruited to date, and re-issuing the invitation to the survey. No further contact occurred with non-responders to this email.

Following recruitment, an independent clinical research administrator (CRA) assigned each panellist a personal identification number (PIN). This PIN was required to access the electronic, web-based surveys. Additionally, the PIN ensured panellist confidentiality when completing the survey, and was used by the CRA when referring any panellist correspondence to the Steering Committee chair.

### Procedure

The Delphi survey comprised three rounds: Round 1 presented an open-ended question, which generated items for quantitative rating in the subsequent two rounds. Additionally, all rounds had provision for general comments regarding the Delphi survey. There was a 5–6 week interval between rounds, for data analysis, survey development, and pilot testing [41]. An independent six-member pilot team tested each round of the survey, to ensure accessibility for panellists who used English as a second language, or who were unfamiliar with electronic surveys [41].

Delphi panellists were asked to complete each round of the survey over a 14-day period. Surveys took 20–40 minutes to complete, could be completed over several sessions, and allowed panellists to review their answers before submitting. Reminders were emailed to non-completers at day 10; additional reminders followed one week and two weeks after the requested submission date. Only panellists who completed a survey round were included in the subsequent round.

#### *Round 1*

The aim of Round 1 was to collect qualitative data on the components individual panellists considered key to yoga interventions for musculoskeletal conditions [41]. Informed consent was firstly obtained, and then demographic information collected, including occupation, research experience, and personal yoga practice of panellists.

Panellists then answered a single open-ended question:

“In my professional opinion, a key component in a yoga intervention protocol for musculoskeletal conditions is…”

Detailed answers were requested. For example, if panellists presented ‘class duration’ as a key component, a numeric duration was required. To increase the breadth and clarity of answers supplied, panellists were required to provide a minimum of six answers [41,57].

#### *Round 2*

The aim of Round 2 was to begin the process of group consensus. The survey consisted of three parts. Part 1 presented a summary of Round 1 results, including panellists’ demographics, and themes generated from the thematic analysis of qualitative data. In Part 2, panellists quantitatively rated items data-driven from analysis of the Round 1 survey, with provision for panellists to provide optional comments on individual items [54]. Part 3 allowed panellists to suggest new items for inclusion in the Round 3 survey, and to make general comments regarding the Delphi process.

When rating the items in Part 2, panellists were asked to:

… consider the importance of the item as a reference tool to assist future researchers in the design and reporting of yoga interventions for musculoskeletal conditions.

Items in Part 2 were grouped according to the theme and subthemes reported in Part 1; each theme beginning with a brief narrative overview of Round 1 comments [58]. The majority of items (referred to henceforth as Likert items) were rated on a 5-point Likert scale, ranging from 1 = “Of no importance” to 5 = “Extremely important”. Due to the diversity of panellists’ expertise, it was expected that not all items would be relevant to all panellists; thus an option to express “No view” was included.

The exception to this form of Likert rating was a five-item subtheme regarding minimum parameter values (Theme 1, subtheme 2; referred to henceforth as parameter items). Each of these items provided 4–5 options (which included specific numeric values and one option of ‘Other’). Panellists were asked to choose which specific option they considered most important (for example, the option of 6, 8, or 12 weeks as a minimum intervention duration), rather than rate the item for overall importance as per the Likert items.

#### *Round 3*

The Round 3 survey followed the same three-part format, thematic grouping, and rating procedure of items as in the previous round. Themes began with a brief narrative overview of qualitative comments from the previous round. Additionally, in Part 2 of the survey each item was presented with a visual (bar graph) and statistical (median and interquartile range (IQR)) summary of its quantitative Round 2 ratings. Panellists were asked to consider, but not be limited by, this feedback when rating each item [58,59].

### Analysis

Analysis of data occurred at the conclusion of each survey round, and informed the inclusion of items for the subsequent round [50,51]. Qualitative data generated from the Round 1 open-ended question were analysed using thematic analysis [60]. Items generated from this analysis were worded using the panellists’ own terms and phrases [41] and grouped into themes for rating in Round 2. Additionally, qualitative data generated from panellist comments were summarised, and reported back to panellists in subsequent rounds of the survey.

Treatment of quantitative data was identical in Round 2 and Round 3 of the survey; with data analysed in Statistical Package for the Social Sciences (SPSS) software, Version 19 [61]. Analysis of Likert items involved calculation of central tendency (median), variance (IQR), and percentage of panellists who rated an item on each point of the scale (including the option of ‘No view’); analysis of parameter items involved calculating the percentage of panellists who voted for each option. These percentage values then determined the consensus level of each item [43].

### Consensus

There are no established consensus criteria for Delphi surveys, with previous studies setting levels ranging from 51-80% [49,51,62]. For the purpose of the current study, the Steering Committee determined *a priori* criteria to develop a list of recommendations containing a limited number of high consensus items, as follows [54,59].

Likert items rated as “Very important” or “Extremely important” by at least 80% of panellists were automatically included in the list of Delphi recommendations. Conversely, items rated “Of no importance” or “Of little importance” by at least 50% of panellists, or rated as “Important”, “Very important”, or “Extremely important” by less than 75% of panellists were automatically excluded from the next survey round. Items rated “Important”, “Very important” or “Extremely important” by at least 75% of panellists were forwarded to the subsequent round for re-rating. Calculation of consensus excluded panellists who chose the “No view” option, in recognition that some items were irrelevant or unfamiliar to them.

Parameter items that received at least 50% of panellists’ votes were automatically included in the Delphi recommendations; remaining items were forwarded to the subsequent round for re-rating. This lower level of consensus for parameter items compared to Likert items was chosen, as it was based on the percentage of votes received for one option (for example, 12 weeks), in contrast to the Likert-based consensus which combined percentages of two to three options (for example “Very important” and “Extremely important”).

Termination criteria for the Delphi survey was based on inter-round stability of non-consensus items at the completion of Round 3 [51]. If the median and IQR of a non-consensus item remained stable or decreased between Rounds 2 and 3, the item was not forwarded to a subsequent round. Conversely, if the median and IQR of an item increased between rounds, then rating of the item in a subsequent round was required.

## Results

**Figure 1:**
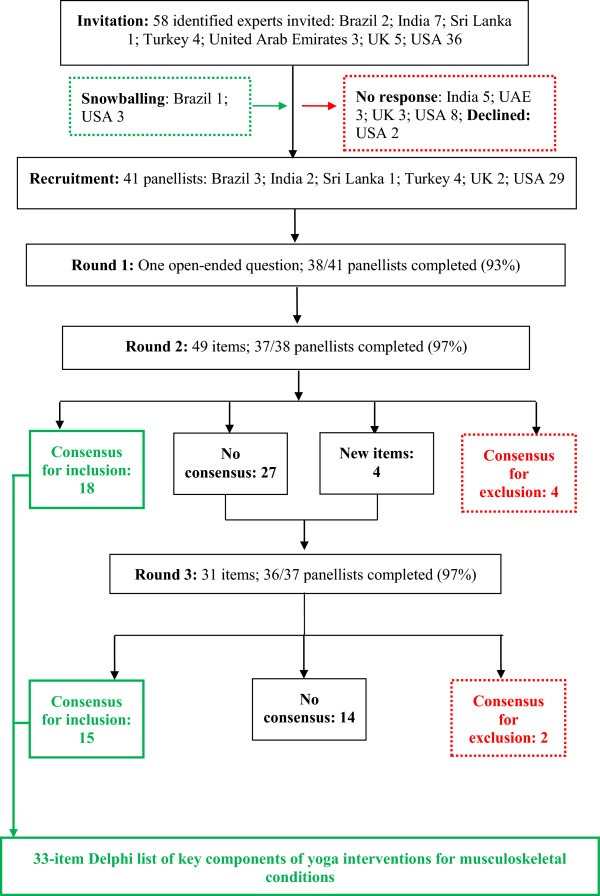
**Flow of panellists and items through the three rounds of the Delphi survey.** UAE: United Arab Emirates; UK: United Kingdom; USA: United States of America.

Figure 1 presents the flow of panellists and items through the Delphi survey. Forty-one panellists were recruited: 37 via direct invitation, and four via snowballing. Of these 41, three (USA) did not access the Round 1 survey; one (India) did not access Round 2, and one (USA) did not access Round 3. The following results section refers to completers only.

### Demographics of survey completers

Thirty-six panellists, representing six countries, completed all three rounds of the Delphi survey (Table 1). On average, panellists had 12 years’ involvement in musculoskeletal research (range 0–40 years), and 9 years involvement in yoga research (range 0–35 years). Twenty panellists identified more than one primary role in yoga for musculoskeletal research, most commonly as both researcher and yoga instructor (36%), or yoga instructor and yoga consultant (14%). Thirty-one of the panellists (86%) had a personal yoga practice, collectively representing 17 different styles or schools of yoga.

**Table 1:** Demographic data of the 36 panellists completing the 3-Round Delphi survey

**Demographic**	**N (%)**
Country of current employment:	
● USA	25 (69)
● Turkey	4 (11)
● Brazil	3 (8)
● UK	2 (6)
● India	1 (3)
● Sri Lanka	1 (3)
Primary occupation:	
● Yoga therapist/instructor	14 (38)
● Researcher/Academic	12 (33)
● Physician	7 (19)
● Physiotherapist	3 (8)
Number of years involved in musculoskeletal research (Mean [SD]):	12 [10]
Number of years involved in yoga research (Mean [SD]):	9 [7]
Primary involvement in yoga research for musculoskeletal conditions*:	
● Researcher	24 (67)
● Yoga instructor	22 (61)
● Private yoga consultant	10 (28)
● Physiotherapist	5 (14)
● Other	2 (6)
Primary musculoskeletal conditions researched with Yoga*:	
● Back pain	13 (36)
● Arthritis	13 (36)
● Fibromyalgia/chronic pain	6 (17)
● Spinal disorders	6 (17)
● Osteoporosis	5 (14)
Number of panellists who personally practice yoga:	31 (86)
Number of years panellists have personally practised yoga (Mean [SD]):	21 [11]
Schools, lineages or styles of yoga practiced by panellists: Ananda; Anusara; Ashtanga; Bihar; Bikram; Classical yoga; Hatha yoga; Integral; Iyengar; Kaivalyadhama; Kripalu; Krishnamacharya; Kundalini; Patanjali’s yoga; Raja yoga; Viniyoga; Vinyasa	

*Panellists could provide more than one response.

### Item generation and rating

#### *Round 1*

Thirty-eight of the 41 recruited panellists completed the Round 1 survey (93%). The single open-ended question generated 331 answers, with an average of 8.7 answers per panellist. Thematic analysis generated 49 items (44 Likert items and five parameter items), which were grouped under five themes and six subthemes for presentation in the Round 2 survey.

The first theme, *“Defining the yoga intervention”* included subthemes of intervention parameters (six items), minimum numeric values of intervention parameters (five items), and appropriateness of the intervention to a musculoskeletal population (five items). The second theme, *“Types of yoga practices”*, comprised eight items of yoga practices for inclusion in intervention protocols. Theme 3, *“Delivery of the yoga protocol”*, included three subthemes of qualities of the yoga instructors (four items), best practice instruction in delivery of the protocol (five items), and resources provided to participants to facilitate their yoga practice (three items). Theme 4, *“Domains of outcome measures”,* presented six items concerning outcome measures panellists suggested as important in yoga interventions for musculoskeletal conditions. The fifth theme, *“Reporting of the yoga intervention”* consisted of seven items relating to the degree of detail necessary for reproducibility of the yoga interventions.

#### *Round 2*

Supplementary analysis data for Round 2 is available in Additional file 1. Thirty-seven of the 38 Round 1 panellists completed the survey (97%). *A priori* consensus for automatic inclusion in the final Delphi recommendations was reached for 17 of the 44 Likert items (consensus range 81-97%) and one of the five parameter items (consensus 54%). Conversely, four Likert items met exclusion criteria and were removed from the survey. The remaining 23 Likert and four parameter items met criteria for forwarding to Round 3. Qualitative analysis of Round 2 comments generated four new Likert-scale items, resulting in a total of 31 items for the Round 3 survey.

#### *Round 3*

Thirty-six of the 37 panellists (97%) completed the survey. Eleven Likert items (consensus range 80-89%) and four parameter items (consensus range 50-61%) reached consensus for inclusion in the Delphi recommendations, and two Likert items met consensus criteria for exclusion (Additional file 2).

The remaining 14 Likert items, including two of the new items, did not reach consensus for inclusion or exclusion. Twelve of these 14 items were analysed for inter-round stability, and did not meet criteria for forwarding to a subsequent round (Additional file 3). The Round 3 ratings of the remaining two new Likert items were compared with those of Round 2 items with similar descriptive statistics, to determine patterns of inter-round stability. Based on this analysis, these two items were not forwarded to a subsequent round.

Qualitative analysis of panellists’ comments generated no new items. Accordingly, the Steering Committee terminated the Delphi survey at the conclusion of Round 3. The Steering Committee was unanimous in accepting the 33-item list of Delphi recommendations of key components for the design and reporting of yoga interventions for musculoskeletal conditions (Table 2).

**Table 2:** Delphi recommendations for the design and reporting of yoga interventions for musculoskeletal conditions

**Theme: *subtheme***	**Item Description**
Defining the yoga intervention:	
● *Types of intervention parameters*	
	1 Dosage of yoga (hours/intervention)
	2 Duration of the yoga intervention (total number of weeks)
	3 Duration of the yoga session (minutes/session)
	4 Frequency of the yoga session (number of sessions/week)
● *Minimum parameter values*	
	5 Recommended minimum duration of a yoga intervention for musculoskeletal conditions: 8 weeks
	6 Recommended minimum duration of a yoga session for musculoskeletal conditions: 60 minutes
	7 Recommended minimum frequency of a yoga session for musculoskeletal conditions: Once per week
	8 Recommended minimum frequency of home practice for musculoskeletal conditions: Three times per week
	9 Recommended minimum session duration of home practice for musculoskeletal conditions: 30 minutes
● *Appropriateness of the intervention to a musculoskeletal population*	
	10 Expectations of study participants (i.e. attendance, abstinence from co-interventions) should be clearly specified prior to recruitment
	11 Yoga practices included in the protocol should be appropriate for the health and fitness limitations of the musculoskeletal conditions being studied
	12 The intervention protocol should allow for modification of yoga practices to accommodate participants individual musculoskeletal limitations
	13 The musculoskeletal condition being researched must be clearly defined
Types of yoga practices to include	
	14 Yoga postures/Asana
	15 Yoga breathing/Pranayama
	16 Yoga relaxation techniques
	17 Mindfulness
Delivery of the yoga protocol:	
● *Yoga instructors*	
	18 Yoga instructors should have a recognised yoga teaching qualification
	19 Yoga instructors should have experience in teaching yoga to people with musculoskeletal conditions
	20 Yoga instructors should be monitored for fidelity of delivery of the yoga intervention
● *Best practice instruction*	
	21 Best practice instruction of a yoga protocol for musculoskeletal conditions should emphasise integration of the yoga practices of body, breath and mind
	22 Best practice instruction of a yoga protocol for musculoskeletal conditions should emphasise principles of safety in carrying out yoga practices
	23 Best practice instruction of a yoga protocol for musculoskeletal conditions should emphasise principles of postural alignment
	24 Best practice instruction of a yoga protocol for musculoskeletal conditions should emphasise principles of integrating yoga practice into study participants’ daily activities
● *Study participant resources*	
	25 Written instructions for home practice
Domains of outcome measures to include	
	26 Outcome measures of physical function
	27 Outcome measures of activities of daily living
	28 Outcome measures of pain
	29 Outcome measures of psychological well-being
	30 Outcome measures of quality of life
	31 Both biomedical and psychosocial outcome measures should be included within an intervention
Reporting of yoga interventions for musculoskeletal conditions	
	32 Accepted reporting guidelines such as the *“CONSORT Statement to Trials of Nonpharmacologic Treatment”* should be followed when reporting a yoga intervention for musculoskeletal conditions
	33 Names of all yoga practices should be clearly detailed in the study write-up

### Panellist feedback on rating of items in the Delphi survey

Panellists endorsed the timeliness and importance of developing recommendations for future researchers of yoga for musculoskeletal conditions. Additionally, they supported the suitability of the Delphi survey as a means of generating these recommendations. A narrative summary of panellists’ comments follows, presented thematically. Direct, verbatim quotes from panellists are presented in italics, to illustrate the diversity of opinions in these themes.

#### *Defining the yoga intervention*

Panellists considered it important to establish minimum parameter values for clinical yoga trials. However, the overall dosage of yoga delivered during the intervention was regarded as being more important than individual parameter values. Panellists stated that the combination of duration and frequency parameters to deliver an intervention dosage was dependent on the demographics of study participants, with one panellist commenting:

Musculo-skeletal conditions are likely to benefit from once- or twice-weekly yoga, whereas if there are also mental health problems (e.g. depression associated with back pain) patients would benefit from daily doses.

Home practice of yoga was the most controversial and polarising issue raised in the Delphi survey. Some panellists considered home practice essential for developing long-term independent practice. Others were strongly opposed to home practice, citing potential safety issues of a yoga-naïve clinical population practising without appropriate supervision. Additionally, panellists stated the difficulty of enforcing and monitoring home practice, due to their experience of low study participant motivation for self-practice.

#### *Content of the yoga protocol*

Broad categories of yoga practices, such as physical and breathing practices, were included as key intervention components. However, panellists considered the choice of specific yoga postures or breathing techniques to be dependent on the study population. Regarding this, the importance of clearly defining the clinical musculoskeletal condition of study participants was emphasised, to enable the choice of safe and appropriate yoga practices.

Panellists emphasised the importance of the non-physical aspects of yoga:

Anyone can do various postures/asanas as a conditioning program. The art of yoga is the facilitation of the parasympathetic system and regulation of the autonomic nervous system. Therefore, these items (meditation and mindfulness) are crucial aspects of the mind-body connection in yoga based interventions.”

Mindfulness was included in the Delphi recommendations; in contrast, there was no consensus regarding the inclusion of meditation. While the majority of panellists rated meditation as important, qualitative comments indicated they viewed meditation as less acceptable or accessible to novice yoga practitioners compared to mindfulness practices. However, panellist comments suggest there may have been confusion over the boundary between mindfulness and meditation. For example, some panellists referred collectively to “mindfulness meditation”, while for others:

My response …assumes that by mindfulness, the survey is referring to one-pointed attention and not the specific meditation technique called “Mindfulness.”

#### *Delivery of the yoga protocol*

Panellists offered a pragmatic perspective on the need for yoga instructors to have specialised teaching qualifications in therapeutic yoga. Concern regarding the safety of study participants taught by inappropriately trained instructors was balanced with acknowledging the current paucity of internationally recognised therapeutic yoga qualifications. As such, many panellists viewed experience as more important than qualification:

New yoga research would be unlikely to happen, unless one allows teachers to teach therapeutically where they have some experience, but not necessarily extra qualifications. After initial professional yoga teacher training (extremely important), experience is more important than qualifications.

The instruction of postural alignment, but not provision of props for class practice (used to improve alignment) was included in the list of Delphi recommendations. Some panellists regarded props as safe to use in a clinical population, while others viewed prop use as associated with certain styles of yoga, such as Iyengar. Many panellists stated a preference for modifying or simplifying postures when teaching a clinical population, rather than over-reliance on use of props.

The area of home practice again polarised opinions. Some panellists felt that providing resources was essential to encourage home practice; while others re-emphasised the potential safety risks of unsupervised self-practice in clinical populations. Suggestions for enhancing safety included providing each study participant with a range of resources based on their preference and learning style. These could include written instructions, photos of yoga postures, and guided meditation CDs.

#### *Domains of outcome measures*

This theme of outcome measures received the lowest number of comments, reflecting the high consensus levels of its six items. Panellists noted that due to the comparatively recent emergence of yoga research:

The more outcome measures the better. We simply don’t know enough about what outcome measures are affected by yoga practice. We need to learn this in part by testing.

#### *Reproducibility of the yoga intervention*

Panellists considered detailed descriptions of yoga interventions to be important for reproducibility. However they also highlighted a tension between standardisation and individualisation, cautioning that too much detail might become both restrictive and prescriptive. For example:

To truly be yoga, there has to be room for responding to the individual needs and limitations of students, which is not possible if it is scripted down to each breath. However, the degree of variability should be described fully.

Panellists commented that journal limits on word counts and tables often restricted the amount of detail reported. Suggestions to improve reproducibility included providing links to supplementary material, and post-trial publication of teaching manuals.

## Discussion

The aim of this Delphi survey was to address issues of heterogeneity associated with yoga interventions for musculoskeletal conditions. Recognised experts from six countries completed three rounds of a web-based survey, in which they first suggested, then rated items they considered as key intervention components. A moderate recruitment rate and high completion rate indicated the timeliness and importance of addressing intervention heterogeneity among these international researchers [41,43,48].

Consensus among the experts resulted in the development of a 33-item list of key components for the design and reporting of yoga interventions for musculoskeletal conditions. Items related to five themes concerning defining the yoga intervention, types of yoga practices to include in an intervention, delivery of the yoga protocol, domains of outcome measures, and reporting of the yoga intervention. These Delphi recommendations offer a non-prescriptive reference tool for future researchers, promoting a level of standardisation across clinical trials. The high consensus levels required for inclusion of an item in the Delphi list provides a clear, united statement from experts regarding the direction of future research. Geographical and occupational diversity of experts ensure these recommendations have international applicability, and encompass all areas of expertise and practise within a research team.

The minimum recommended parameters of a weekly, 60-minute yoga class reflect a common pattern in trials of yoga for low back pain (LBP) [63-67]. Additionally, despite polarised views regarding home practice, the majority consensus of a minimum 30 minutes of practice, three times per week is also reflective of previous musculoskeletal interventions [67-72]. However, the Delphi recommendation of a minimum 8-week intervention results in a minimum intervention dosage of 8 hours of instructor-led yoga. This is well below the mean intervention dosage of 29 hours of instructor-led yoga identified in a review of clinical trials of yoga for musculoskeletal conditions [31]; and below an average dosage of 20 hours in exercise-based therapies for LBP [73].

Some Delphi recommendations, such as monitoring of instructor fidelity, reflect current best practice guidelines for health behaviour interventions [74]. However, not all aspects of intervention design and reporting were considered amenable to standardisation. For example, experts rated class size as important, but commented this was dependent on the study population. Therefore, classes could not be standardised in isolation of other factors such as functional limitations of study participants. Similar comments were received regarding other items excluded from the survey. These opinions clearly indicated that experts considered it important to retain a pragmatic balance between such issues as standardisation versus individual study participant needs, and ideal study design versus time and budget constraints.

Limitations to the current study are noted. Firstly, the views of Delphi panellists may differ from those experts who declined participation, and may therefore not adequately represent experts in the field of interest [41,42,49]. To minimise this limitation, a comprehensive recruitment process involving a systematic review and snowball technique was used, to ensure a representative range of international researchers and yoga consultants involved in the field were invited to participate in the survey [48,55]. Additionally, as the majority of experts who accepted the invitation to participate in the Delphi process were from the USA and Europe, the Delphi recommendations may incorporate a Western perspective less relevant to non-Western researchers. The reasons for declining to participate are unclear; however, it is possible that the method of email invitation may not be an effective or appropriate form of recruitment for some countries.

Future research in the development of these Delphi recommendations will involve clearly defining the boundaries of each item. For example, does an intervention dosage of yoga include home practice; how would yoga instructors be monitored for fidelity; and in what language would the names of yoga practises be reported in the study write-up? Additionally, unclear item definition identified by panellists, such as differentiating between the practices of mindfulness versus mindfulness meditation, require further clarification. Given the positive response of experts to the use of the Delphi survey, this method is suggested as a means to further develop and define these Delphi recommendations. Additional recruitment methods, such as telephone contact, may enhance international interest in the project.

## Conclusion

The current study aimed to address issues of heterogeneity associated with yoga interventions for musculoskeletal conditions. The resultant Delphi recommendations, based on expert consensus, provides 33 items related to defining the yoga intervention, types of yoga practices to include, delivery of the yoga protocol, domains of outcome measures, and reporting of the yoga intervention to consider when designing a yoga protocol for musculoskeletal conditions. It is anticipated future research incorporating the Delphi guidelines will facilitate high quality international research in the field of yoga for musculoskeletal conditions, increase homogeneity of intervention components and parameters, and enhance the comparison and reproducibility of research in this field.

## Competing interests

The authors declare that they have no competing interests.

## Authors’ contributions

All authors were involved in the conceptualisation and design of the study, and panellist recruitment. LW conducted the study and drafted the manuscript. LW, SS, and GDB contributed to data analysis and survey development. All authors were involved in manuscript revisions, and read and approved the final manuscript.

## Pre-publication history

The pre-publication history for this paper can be accessed here:

http://www.biomedcentral.com/1472-6882/14/196/prepub

## Supplementary Material

Additional file 1 **Summary of Round 2 quantitative analysis of the Delphi survey.** This file presents a summary of the quantitative analysis of the 44 Likert items and the five parameter items from Round 2 of the Delphi survey. Click here for file

Additional file 2 **Summary of Round 3 quantitative analysis of the Delphi survey.** This file presents a summary of the quantitative analysis of the 27 Likert items and the four parameter items from Round 2 of the Delphi survey. Click here for file

Additional file 3 **Summary of inter-round stability analysis of non-consensus items of the Delphi survey.** This file presents a summary of the inter-round stability analysis of 12 items that did not reach consensus in both Round 2 and Round 3. Click here for file
