# Supplementary material for: Establishing key components of yoga interventions for musculoskeletal conditions: a Delphi survey
Source: BMC Complement Altern Med. 2014 Jun 18;14:196. doi: 10.1186/1472-6882-14-196 (PMC4081491; doi:10.1186/1472-6882-14-196)
Supplement: Additional file 1 — Summary of Round 2 quantitative analysis of the Delphi survey. This file presents a summary of the quantitative analysis of the 44 Likert items and the five parameter items from Round 2 of the Delphi survey. [file 1472-6882-14-196-S1.pdf]

Additional file 1. Summary of Round 2 quantitative analysis of the Delphi survey

Table 1.1. Summary of quantitative analysis of the 44 Likert items

Item	Likert rating (%)*							M [IQR]	Outcome
	1	2	3	4	5	No view	4+5		
**THEME 1: Defining the yoga intervention**									
Subtheme 1: Types of intervention parameters									
• Duration of the yoga intervention	0	0	16	46	38	0	84	4 [4,5]	Included
• Duration of the yoga session	0	3	32	46	19	0	65	4 [4,4]	Forwarded
• Frequency of the yoga session	0	0	24	54	22	0	76	4 [4,4]	Forwarded
• Class size	0	3	62	19	16	0	35	3 [3,4]	Forwarded
• Frequency of home practice	0	11	31	25	33	3	58	4 [3,5]	Forwarded
• Duration of home practice	0	17	47	22	14	3	36	3 [3,4]	Forwarded
Subtheme 3: Appropriateness of the intervention									
• The musculoskeletal condition being researched must be clearly defined	0	0	22	27	51	0	78	5 [4,5]	Forwarded
• The study population should be homogenous in their physical ability to carry out the protocol	0	28	42	11	19	3	30	3 [2,4]	Excluded
• Expectations of study participants should be clearly specified prior to recruitment	6	0	14	25	56	3	81	5 [4,5]	Included
• Yoga practices should be appropriate for the health and fitness limitations of the musculoskeletal condition	3	0	5	14	78	0	92	5 [5,5]	Included
• Protocol should allow for modification of yoga practices to accommodate individual limitations	0	3	6	22	69	3	91	5 [4,5]	Included

**THEME 2: Types of yoga practices**									
• Yoga postures/Asana	0	0	9	26	66	5	92	5 [4,5]	Included
• Yoga breathing/Pranayama	0	3	15	29	53	8	82	4 [4,5]	Included
• Yoga relaxation techniques	0	0	17	36	47	3	83	4 [4,5]	Included
• Meditation	0	17	42	28	14	3	42	3 [3,4]	Forwarded
• Mindfulness	0	8	19	31	42	3	73	4 [3,5]	Forwarded
• Yoga philosophy	0	36	33	19	11	3	30	3 [2,4]	Excluded
• Yoga Nidra	3	43	40	7	7	19	14	2 [2,3]	Excluded
• Yoga chanting	18	64	12	6	0	11	6	2 [1,2]	Excluded
**THEME 3: Delivery of the yoga protocol**									
Subtheme 1: Yoga instructors									
• Yoga instructors should have a recognised yoga teaching qualification	3	5	8	24	60	0	84	5 [4,5]	Included
• Yoga instructors should have a specialised qualification in therapeutic yoga	6	6	25	25	39	3	64	4 [3,5]	Forwarded
• Yoga instructors should have experience in teaching yoga to people with a musculoskeletal condition	3	0	14	25	58	3	83	5 [4,5]	Included
• Yoga instructors should be monitored for fidelity of delivery of the yoga intervention	0	8	22	24	46	0	70	4 [3,5]	Forwarded
Subtheme 2: Best practice in delivery of the protocol									
• Best practice instruction should emphasise difference between yoga and physical therapy exercises	0	25	31	25	19	3	44	3 [2,4]	Forwarded
• Best practice instruction should emphasise integrating yoga practices of body, breath and mind	0	0	14	31	56	3	87	5 [4,5]	Included

• Best practice instruction should emphasise principles of safety in carrying out yoga practices	0	0	3	19	78	3	97	5 [4,5]	Included
• Best practice instruction should emphasise principles of postural alignment	0	12	15	15	59	8	74	5 [3,5]	Forwarded
• Best practice instruction should emphasise principles of integrating yoga into daily activities	0	6	19	36	39	3	75	4 [3,5]	Forwarded
Subtheme 3: Participant resources									
• Props for class practice	0	15	29	32	24	8	56	4 [3,4]	Forwarded
• Written instructions for home practice	0	6	17	39	39	3	78	4 [3,5]	Forwarded
• Audio-visual aids (CD, DVD) for home practice	0	14	31	33	22	3	55	4 [3,4]	Forwarded
**THEME 4: Domains of outcomes measures**									
• Physical function	0	5	14	30	51	0	81	5 [4,5]	Included
• Activities of daily living	0	0	16	27	57	0	84	5 [4,5]	Included
• Pain	0	0	14	19	68	0	87	5 [4,5]	Included
• Psychological well-being	0	0	8	32	60	0	92	5 [4,5]	Included
• Quality of life	0	0	5	30	65	0	95	5 [4,5]	Included
• Both biomedical and psychosocial outcome measures should be included within an intervention	0	0	31	25	44	3	69	4 [3,5]	Forwarded
**THEME 5: Reporting of the yoga intervention**									
• Accepted guidelines (e.g. CONSORT) should be followed when reporting yoga interventions	0	3	34	22	41	14	63	4 [3,5]	Forwarded
• Names of all yoga practices should be clearly detailed in the study write-up	0	3	16	27	54	0	81	5 [4,5]	Included
• Duration of yoga practices should be clearly detailed in the study write-up	0	16	16	30	38	0	68	4 [3,5]	Forwarded

• The purpose of the yoga practices should be clearly detailed in the study write-up	0	5	27	32	35	0	67	4 [3,5]	Forwarded
• Visual descriptions of yoga practices should be provided in study write-up or supplementary document	0	8	38	35	19	0	54	4 [3,4]	Forwarded
• Sequencing of yoga practices over duration of intervention should be clearly detailed in study write-up	0	3	30	38	30	0	68	4 [3,5]	Forwarded
• Parameters of protocol modification should be clearly detailed in the study write-up	0	3	35	38	24	0	62	4 [3,5]	Forwarded

Symbols: *: 1= “Of no importance”, 2= “Of little importance”, 3= “Important”, 4= “Very important”, 5= “Extremely important”. Calculation of consensus for Likert ratings 1-5 excludes panellists who chose the “No view” option; whereas the percentage of “No view” items are based on the total number of panellists in Round 2. **Abbreviations:** IQR: interquartile range; M: median

Table 1.2. Summary of quantitative analysis of the five parameter items

Theme 1, Subtheme 2: Defining the yoga intervention, Minimum values of parameters	Option (% panellists choosing this option)					Outcome
What is a recommended MINIMUM duration of a yoga intervention for musculoskeletal conditions?		4 weeks (19%)	8 weeks (49%)	12 weeks (22%)	Other (11%)	Forwarded
What is a recommended MINIMUM duration of a yoga session for musculoskeletal conditions?	45 minutes (19%)	60 minutes (54%)	75 minutes (11%)	90 minutes (5%)	Other (11%)	Included
What is a recommended MINIMUM frequency of a yoga session for musculoskeletal conditions?		Once/week (41%)	Twice/week (30%)	Three/week (19%)	Other (11%)	Forwarded
What is a recommended MINIMUM frequency of home practice for musculoskeletal conditions?		Three/week (46%)	Five/week (16%)	Days of no class practice (16%)	Other (22%)	Forwarded
What is a recommended MINIMUM session duration of home practice for musculoskeletal conditions?		10 minutes (16%)	20 minutes (46%)	30 minutes (27%)	Other (11%)	Forwarded
