# Supplementary material for: Establishing key components of yoga interventions for musculoskeletal conditions: a Delphi survey
Source: BMC Complement Altern Med. 2014 Jun 18;14:196. doi: 10.1186/1472-6882-14-196 (PMC4081491; doi:10.1186/1472-6882-14-196)
Supplement: Additional file 2 — Summary of Round 3 quantitative analysis of the Delphi survey. This file presents a summary of the quantitative analysis of the 27 Likert items and the four parameter items from Round 2 of the Delphi survey. [file 1472-6882-14-196-S2.pdf]

Additional file 2. Summary of Round 3 quantitative analysis of the Delphi survey.

Table 2.1. Summary of quantitative analysis of the 27 Likert items

**Item**	**Likert rating (%)***							**M [IQR]**	**Outcome**
	**1**	**2**	**3**	**4**	**5**	**No view**	**4+5**		
**THEME 1: Defining the yoga intervention**									
Subtheme 1: Types of intervention parameters									
Dosage of the yoga intervention	0	0	19	50	31	0	81	4 [4,5]	Included †
Duration of the yoga session	0	0	17	75	8	0	83	4 [4,4]	Included
Frequency of the yoga session	0	3	17	67	14	0	81	4 [4,4]	Included
Class size	0	8	61	22	8	0	30	3 [3,4]	No consensus
Frequency of home practice	0	3	46	37	14	3	51	4 [3,4]	No consensus
Duration of home practice	0	6	69	23	3	3	26	3 [3,4]	No consensus
Subtheme 3: Appropriateness of the intervention									
The musculoskeletal condition being researched must be clearly defined	0	0	11	17	72	0	89	5 [4,5]	Included
**THEME 2: Types of yoga practices**									
Meditation	0	18	52	27	3	8	30	3 [2,4]	No consensus
Mindfulness	3	3	12	62	21	6	83	4 [4,4]	Included
**THEME 3: Delivery of the yoga protocol**									
Subtheme 1: Yoga instructors									
Yoga instructors should have a specialised qualification in therapeutic yoga	3	9	11	49	29	3	78	4 [3,5]	No consensus
Yoga instructors should be monitored for fidelity of delivery of the yoga intervention	0	3	17	43	37	3	80	4 [4,5]	Included

Subtheme 2: Best practice in delivery of the protocol									
Best practice instruction should emphasise difference between yoga and physical therapy exercises	3	36	33	22	6	0	28	3 [2,4]	Excluded
Best practice instruction should emphasise the difference between yoga as a practice and yoga as therapy	0	33	49	12	6	8	18	3 [2,3]	Excluded†
Best practice instruction should emphasise principles of postural alignment	0	9	6	18	68	6	86	5 [4,5]	Included
Best practice instruction should emphasise principles of integrating yoga into daily activities	0	3	17	56	25	0	81	4 [4,5]	Included
Subtheme 3: Participant resources									
Props for class practice	0	9	27	42	21	8	63	4 [3,4]	No consensus
Written instructions for home practice	6	6	9	51	29	3	80	4 [4,5]	Included
Audio-visual aids (CD, DVD) for home practice	3	9	26	43	20	3	63	4 [4,5]	No consensus
**THEME 4: Domains of outcomes measures**									
Both biomedical and psychosocial outcome measures should be included within an intervention	0	0	17	49	34	3	83	4 [4,5]	Included
**THEME 5: Reporting of the yoga intervention**									
Accepted guidelines (e.g. CONSORT) should be followed when reporting yoga interventions	0	0	19	56	25	11	81	4 [3,4]	Included
Yoga qualifications and teaching experience of the yoga instructors should be clearly detailed in the study write-up	0	3	31	44	22	0	66	4 [3,4]	No consensus†
Duration of yoga practices should be clearly detailed in the study write-up	3	8	17	53	19	0	72	4 [3,4]	No consensus
The purpose of the yoga practices should be clearly detailed in the study write-up	0	3	26	34	37	3	71	4 [3,5]	No consensus
Visual descriptions of yoga practices should be provided in study write-up or supplementary document	0	3	25	53	19	0	72	4 [3,4]	No consensus
Sequencing of yoga practices over duration of intervention should be clearly detailed in study write-up	0	3	22	39	36	0	75	4 [3,5]	No consensus

Parameters of protocol modification should be clearly detailed in the study write-up	0	3	31	50	17	0	67	4 [3,4]	No consensus
The context of the yoga sessions should be clearly detailed	0	17	57	23	3	3	26	3 [3,4]	No consensus†

Symbols: *: 1= "Of no importance", 2= "Of little importance", 3= "Important", 4= "Very important", 5= "Extremely important". Calculation of consensus for Likert ratings 1-5 excludes panellists who chose the "No view" option; whereas the percentage of "No view" items are based on the total number of panellists in Round 3. †: New item. **Abbreviations**: IQR: interquartile range; M: median

Table 2.2. Summary of quantitative analysis of the four parameter items

Theme 1, Subtheme 2: Defining the yoga intervention; Minimum values of parameters	Option (% panellists choosing this option)					Outcome
What is a recommended MINIMUM duration of a yoga intervention for musculoskeletal conditions?	4 weeks (8%)	6 weeks† (19%)	8 weeks (56%)	12 weeks (11%)	Other (6%)	Included
What is a recommended MINIMUM frequency of a yoga session for musculoskeletal conditions?		Once/week (50%)	Twice/week (33%)	Three/week (8%)	Other (8%)	Included
What is a recommended MINIMUM frequency of home practice for musculoskeletal conditions?		Three/week (61%)	Five/week (11%)	Days of no class practice (3%)	Other (25%)	Included
What is a recommended MINIMUM session duration of home practice for musculoskeletal conditions?		10 minutes (14%)	20 minutes (11%)	30 minutes (61%)	Other (14%)	Included

Symbol: † New option
