# Supplementary material for: Establishing key components of yoga interventions for musculoskeletal conditions: a Delphi survey
Source: BMC Complement Altern Med. 2014 Jun 18;14:196. doi: 10.1186/1472-6882-14-196 (PMC4081491; doi:10.1186/1472-6882-14-196)
Supplement: Additional file 3 — Summary of inter-round stability analysis of non-consensus items of the Delphi survey. This file presents a summary of the inter-round stability analysis of 12 items that did not reach consensus in both Round 2 and Round 3. [file 1472-6882-14-196-S3.pdf]

Additional file 3. Summary of inter-round stability analysis of non-consensus items of the Delphi survey

Item	Round	Likert rating (%)*						M [IQR]	Stability
		1	2	3	4	5	No view		
**THEME 1: Defining the yoga intervention**									
Subtheme 1: Types of intervention parameters									
Class size (number of participants/teacher):	2	0	3	63	19	16	0	3 [3,4]	Rating converged to important; stable median and IQR
	3	0	8	61	22	8	0	3 [3,4]	
Frequency of home practice (number of sessions/week)	2	0	11	31	25	33	3	4 [3,5]	Rating converged to important; stable median decreased IQR
	3	0	3	46	37	14	3	4 [3,4]	
Duration of home practice (minutes/session):	2	0	17	47	22	14	3	3 [3,4]	Rating converged to important; stable median and IQR
	3	0	6	69	23	3	3	3 [3,4]	
**THEME 2: Types of yoga practices**									
Meditation	2	0	17	42	28	14	3	3 [3,4]	Rating converged to important; stable median, decreased IQR
	3	0	18	52	27	3	8	3 [2,4]	
**THEME 3: Delivery of the yoga protocol**									
Subtheme 1: Yoga instructors									
Yoga instructors should have a specialised qualification in therapeutic yoga	2	6	6	25	25	39	3	4 [3,5]	Decreased majority rating from extremely to very important; stable median and IQR
	3	3	9	11	49	29	3	4 [3,5]	
Subtheme 3: Participant resources									
Props for class practice	2	0	15	29	32	24	8	4 [3,4]	Increased rating of very important; stable median and IQR
	3	0	9	27	42	21	8	4 [3,4]	
Audio-visual aids (CD, DVD) for home practice	2	0	14	31	33	22	3	4 [3,4]	

	3	3	9	26	43	20	3	4 [3,4]	Increased rating of very important; stable median and IQR
**THEME 5: Reporting of the yoga intervention**									
Duration of yoga practices should be clearly detailed in the study write-up	2	0	16	16	30	38	0	4 [3,5]	Decreased rating from extremely to very important; stable median, decreased IQR
	3	3	8	17	53	19	0	4 [3,4]	
The purpose of the yoga practices should be clearly detailed in the study write-up	2	0	5	27	32	35	0	4 [3,5]	Stable ratings; stable median and IQR
	3	0	3	26	34	37	3	4 [3,5]	
Visual descriptions of yoga practices should be provided in study write-up or supplementary document	2	0	8	38	35	19	0	4 [3,4]	Increased rating of very important; stable median and IQR
	3	0	3	25	53	19	0	4 [3,4]	
Sequencing of yoga practices over the duration of the intervention should be clearly detailed in the study write-up	2	0	3	30	38	30	0	4 [3,5]	Increased rating of extremely important; stable median and IQR
	3	0	3	22	39	36	0	4 [3,5]	
Parameters of protocol modification (including how props were used) should be clearly detailed in the study write-up	2	0	3	35	38	24	0	4 [3,5]	Ratings converging to very important; stable median, decreased IQR
	3	0	3	31	50	17	0	4 [3,4]	

Symbols: *: 1= "Of no importance", 2= "Of little importance", 3= "Important", 4= "Very important", 5= "Extremely important". Calculation of consensus for Likert ratings 1-5 exclude panelists who chose the "No view" option; whereas the percentage of "No view" items are based on the total number of panellists in each round. **Abbreviations**: IQR: interquartile range; M: median
